# Fourier-Transform Infrared Spectral Library of MXenes

**DOI:** 10.1021/acs.chemmater.4c01536

**Published:** 2024-08-21

**Authors:** Tetiana Parker, Danzhen Zhang, David Bugallo, Kateryna Shevchuk, Marley Downes, Geetha Valurouthu, Alex Inman, Benjamin Chacon, Teng Zhang, Christopher E. Shuck, Yong-Jie Hu, Yury Gogotsi

**Affiliations:** † A.J. Drexel Nanomaterials Institute, Drexel University, 3141 Chestnut St., Philadelphia, Pennsylvania 19104, United States; ‡ Department of Material Science and Engineering, Drexel University, 3141 Chestnut St., Philadelphia, Pennsylvania 19104, United States; § Centro de Investigación en Química Biolóxica e Materiais Moleculares (CIQUS), Universidade de Santiago de Compostela, 15782 Santiago, Spain; ∥ Department of Chemistry and Chemical Biology, Rutgers University, Piscataway, New Jersey 08854, United States

## Abstract

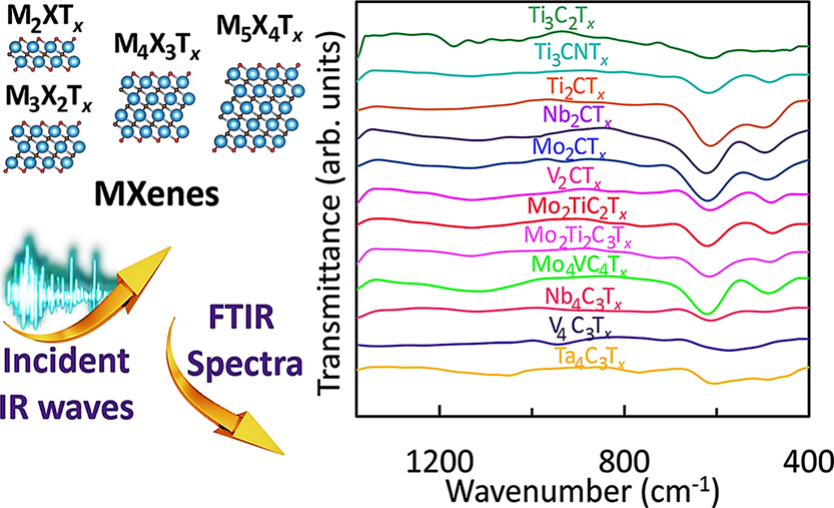

Fourier-transform infrared (FTIR) spectroscopy characterization is a powerful and easy-to-use technique frequently employed for the characterization and fingerprinting of materials. Although MXenes are a large and fastest growing family of inorganic 2D materials, the lack of systematic FTIR spectroscopy studies hinders its application to MXenes and often leads to misinterpretation of the results. In this study, we report experimental and calculated FTIR spectra of 12 most typical carbide and carbonitride MXenes with different compositions (5 transition metals) and all four basic structures, including Ti_2_CT_*x*_, Nb_2_CT_*x*_, Mo_2_CT_*x*_, V_2_CT_*x*_, Ti_3_C_2_T_*x*_, Ti_3_CNT_*x*_, Mo_2_TiC_2_T_*x*_, Mo_2_Ti_2_C_3_T_*x*_, Nb_4_C_3_T_*x*_, V_4_C_3_T_*x*_, Ta_4_C_3_T_*x*_, and Mo_4_VC_4_T_*x*_. The measurements were performed on delaminated MXene flakes incorporated in KBr pellets in the 4000–400 cm^–1^ range. We provide detailed instructions for sample preparation, data collection, and interpretation of FTIR spectra of MXenes. Background correction and spectra smoothing are applied to obtain clear FTIR peaks corresponding to bond vibrations in MXenes. Density functional theory calculations were used for the precise assignment of all characteristic FTIR peaks and an in-depth analysis of the vibration modes. This work aims to provide the 2D material community with the FTIR spectroscopy technique as a reliable method for identifying and analyzing MXenes.

## Introduction

Fourier-transform infrared (FTIR) spectroscopy, a widely used vibrational spectroscopy technique, contributes significantly to the overall scope of material chemistry research. This technique can bring unique insights into 2D materials’ chemical characterization. MXenes, an emerging class of 2D materials reported in 2011,^1^ have attracted much attention from the research community^2^ due to their variety of structures,^3^ abundant redox sites,^4^ metallic conductivity,^5^ hydrophilicity,^6^ and ease of processability.^7^ These properties make them attractive for applications in electrochemical energy storage,^8^ electromagnetic interference shielding,^9^ thermal management,^10^ etc. The MXene structure can be represented by the formula M_*n*+1_X_*n*_T_*x*_, where *n* is an integer from 1 to 4,^11^ M is an early transition metal, X is either carbon (C) or nitrogen (N), and T_*x*_ represents surface terminations (typically, for the wet-chemical top-down route, −F, =O, −OH). Increased interest in MXenes requires characterization techniques that can identify the basic MX structure and account for surface terminations and intercalated species. Greater insights into MXenes chemistry (changes in composition, bonding, structure, etc.) will bring improvement in material quality and broaden the range of MXene applications.

Raman spectroscopy is a well-established technique for MXene characterization.^12,13^ FTIR and Raman techniques are the most widely used types of vibrational spectroscopies. FTIR spectroscopy complements Raman measurements because some Raman-inactive vibration modes, such as E_u_ and A_2u_, may be active in FTIR. This allows for better detection of surface terminations and chemical bonds, which is beneficial for the characterization of MXene organic hybrids,^14^ hydrogen bonding,^15^ etc. Raman spectroscopy, which requires the use of lasers, may often lead to fast heating and material damage. It is particularly important for MXenes, which are highly efficient light-to-heat converters. Despite the existing depth of development in FTIR spectroscopy methods, studies utilizing FTIR spectroscopy of MXenes as an additional characterization technique provide limited insights into FTIR spectrum features^16−22^ and minimal parametric data.^23,24^ There is no FTIR spectroscopical characterization of the MXenes family as a whole (unlike other common materials, including polymers, hydrogels, etc.).^25,26^ Due to the lack of references in this area, misinterpretation and term misuse^27^ are pretty common.

The goal of this work is to establish an inclusive FTIR spectral library for the acceleration of research on MXenes.^1,2^ To complete this goal, we provide recommendations for sample preparation, data collection, and FTIR peaks and vibration modes assignment of 12 selected MXenes: Ti_2_CT_*x*_, Nb_2_CT_*x*_, Mo_2_CT_*x*_, V_2_CT_*x*_, Ti_3_C_2_T_*x*_, Ti_3_CNT_*x*_, Mo_2_TiC_2_T_*x*_, Mo_2_Ti_2_C_3_T_*x*_, Nb_4_C_3_T_*x*_, V_4_C_3_T_*x*_, Ta_4_C_3_T_*x*_, and Mo_4_VC_4_T_*x*_. Furthermore, the theoretical IR vibration modes are predicted via first-principles calculations based on density functional theory (DFT) for the same MXene chemistries with −F and =O surface terminations. FTIR and DFT studies are accompanied by additional X-ray diffraction (XRD) characterization and optical imaging. This work provides the comprehensive data needed for implementing FTIR spectroscopy in the analysis of MXenes.

## Results and Discussion

**Figure 1:**
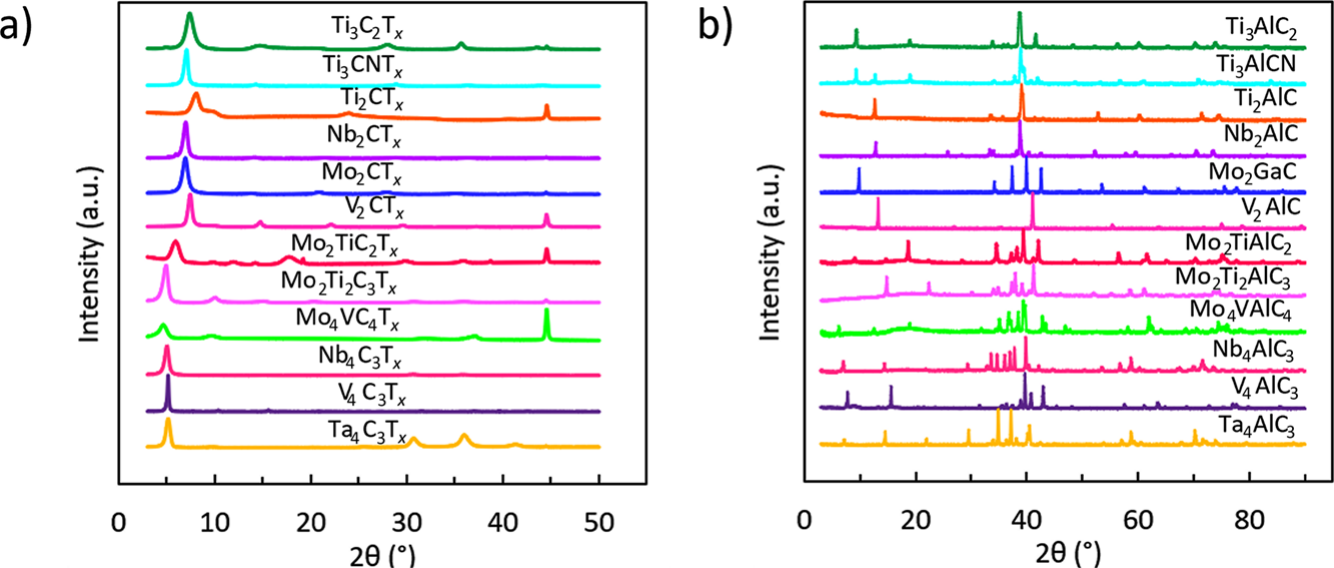
X-ray diffraction (XRD) characterization of MXenes used in this study. (a) XRD patterns of MXenes. (b) XRD patterns of the corresponding precursor MAX phases.

The MXenes were synthesized using a well-established^28,29^ wet-chemical selective etching of corresponding MAX phases and subsequent delamination of multilayer particles,^5,10,30−34^ resulting in the −F, =O, and −OH surface terminations. The synthesis of MXenes and their precursor MAX phases was confirmed by XRD. In Figure 1a, the shift toward lower 2θ and broadening of the (002) peak indicate the conversion of the MAX phases to MXenes. Ti_3_C_2_T_*x*_, Ti_2_CT_*x*_, Mo_2_CT_*x*_, V_2_CT_*x*_, Mo_2_TiC_2_T_*x*_, Mo_2_Ti_2_C_3_T_*x*_, and Mo_4_VC_4_T_*x*_ show minor impurities (traces of cubic carbides or unreacted MAX). XRD also reveals (312) and (211) peaks in Ti_3_AlCN and trace amounts of Mo_2_Ti_2_AlC_3_ in Mo_2_TiAlC_2_. Single (002) peaks in the XRD patterns of MAX phases indicate phase purity of precursors (Figure 1b).^35^

**Figure 2:**
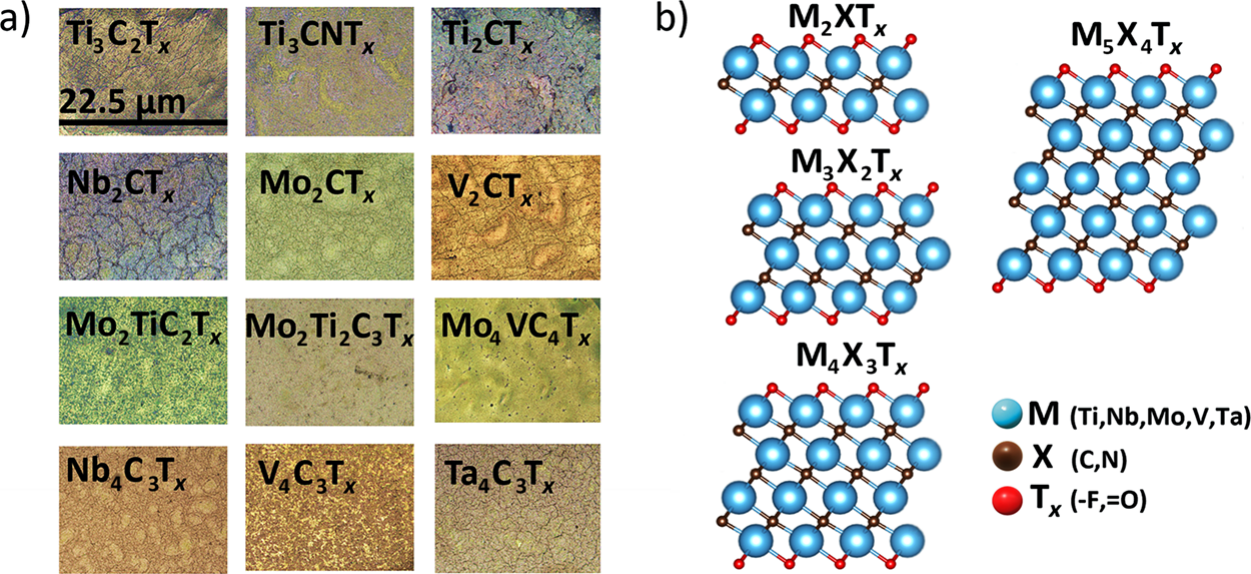
Optical micrographs and structures of MXenes. (a) KEYENCE optical images of MXenes with a 5:1 zoom ratio, where the width of each image is 22.5 μm. (b) MXene chemical structures with different numbers of layers (M_*n*+1_X_*n*_T_*x*_ where *n* is an integer 1–4), where M is a transition metal, X is either carbon (C) or nitrogen (N), and T_*x*_ represents surface terminations (in this work, it is either −F or =O). Images of the molecular structures were generated in VESTA.

**Figure 3:**
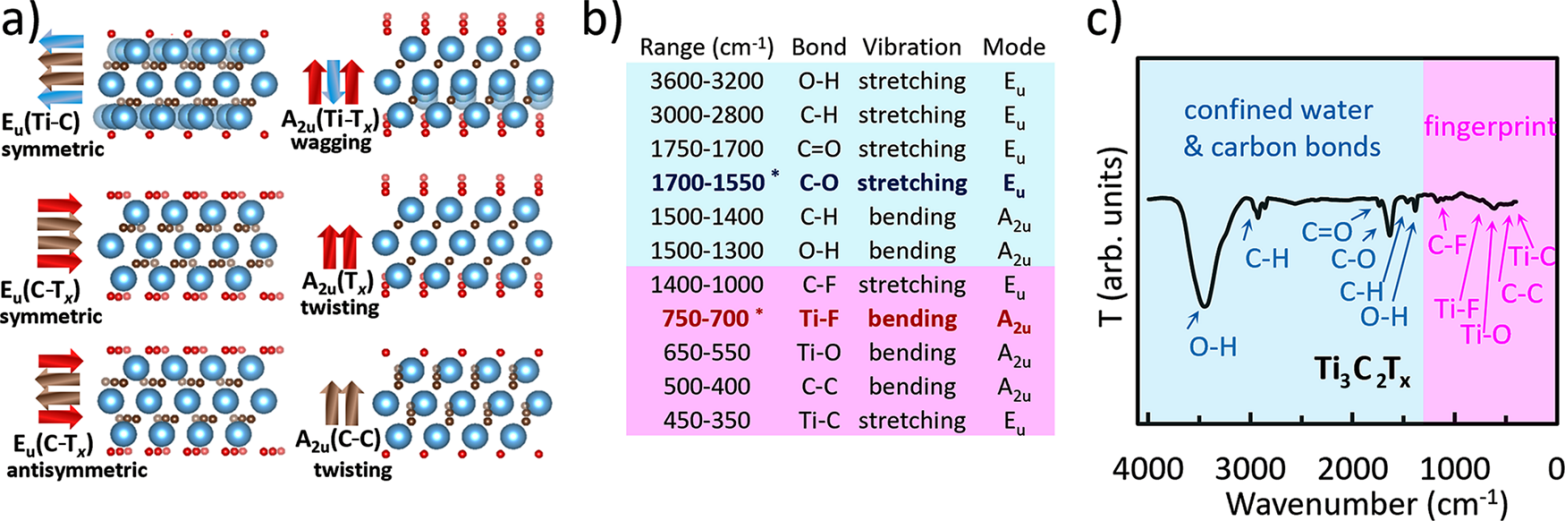
FTIR spectroscopy characterization of Ti_3_C_2_T_*x*_ MXene. (a) E_u_ and A_2u_ vibration modes (the images of molecular structures were generated in VESTA),^200^ (b) FTIR peak positions, and (c) FTIR spectrum with assigned bond vibrations. Note**, the C–O peak undergoes a blue shift from its regular range of 1300–1100 cm^–1^ due to confined water, and the Ti–F peak undergoes a red shift from its regular range of 1000–850 cm^–1^ due to interaction of −F, =O, and Ti atoms simultaneously present in the surface layer. T is transmittance.

**Figure 4:**
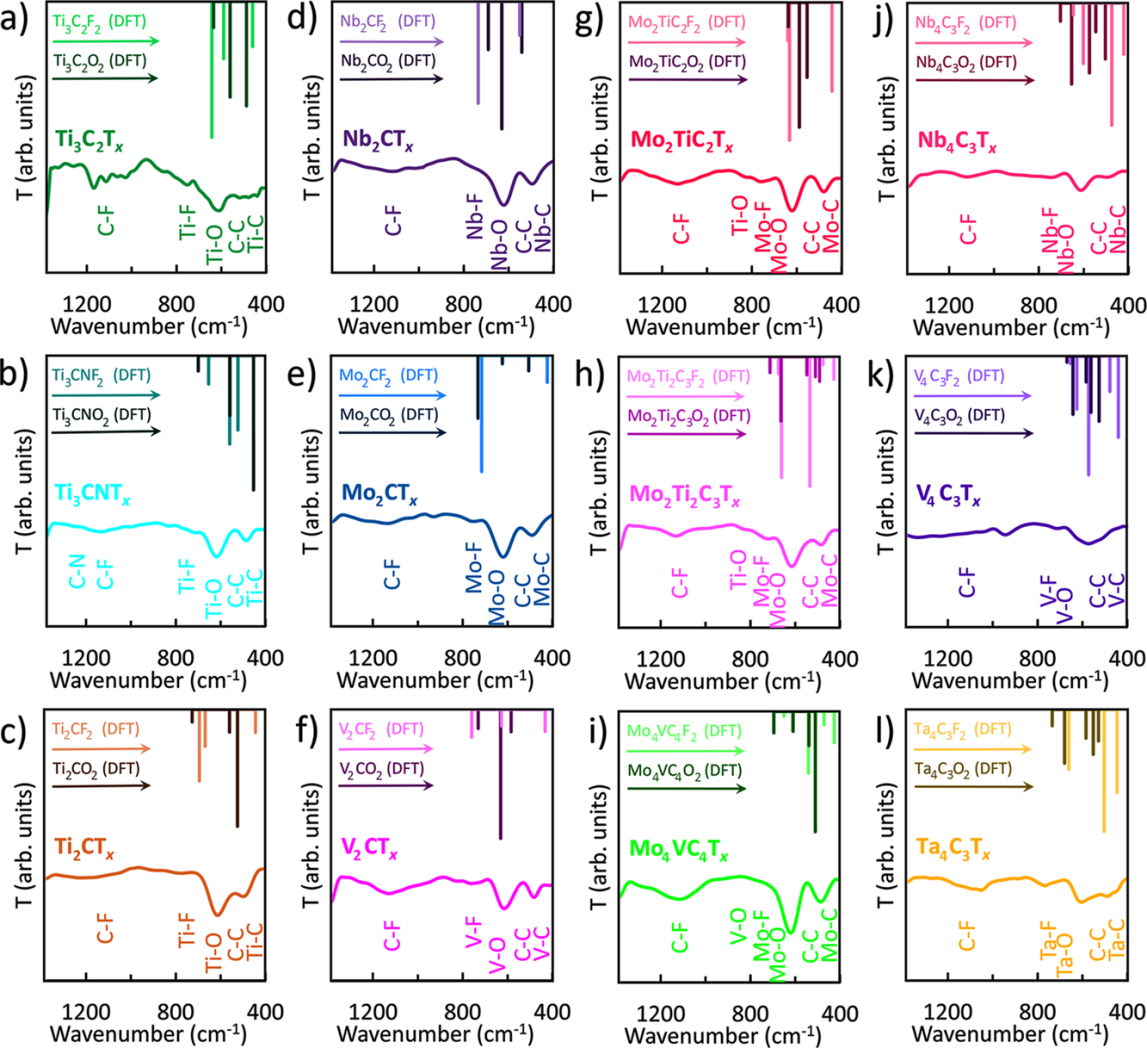
DFT-predicted (−F and =O terminated structures) and measured FTIR spectra of MXenes (mixed T_*x*_ terminations) with bond vibrations assigned; T is transmittance. (a) Ti_3_C_2_T_*x*_, (b) Ti_3_CNT_*x*_, (c) Ti_2_CT_*x*_, (d) Nb_2_CT_*x*_, (e) Mo_2_CT_*x*_, (f) V_2_CT_*x*_, (g) Mo_2_TiC_2_T_*x*_, (h) Mo_2_Ti_2_C_3_T_*x*_, (i) Mo_4_VC_4_T_*x*_, (j) Nb_4_C_3_T_*x*_, (k) V_4_C_3_T_*x*_, and (l) Ta_4_C_3_T_*x*_.

In optical images (Figure 2a), MXenes exhibit vibrant colors, an intrinsic MXene feature.^36^ These colors reflect the difference in the optical properties of MXenes. Figure 2b shows structures of MXenes (M_*n*+1_X_*n*_T_*x*_, with *n* integer from 1 to 4, i.e., M_2_XT_*x*_, M_3_X_2_T_*x*_, M_4_X_3_T_*x*_, and M_5_X_4_T_*x*_). The variety of structure types in MXenes yields great variations in their chemical fingerprints (Figures 3 and 4). For this initial study, ordered crystal structures and single-species surface terminations were chosen for DFT calculations, where T_*x*_ is either −F or =O. Effects of mixed terminations and variation in terminations^37,38^ (−OH, −Cl, −Br, −I, etc.) are left for future studies.

Following successful synthesis and XRD characterization, Ti_2_CT_*x*_, Nb_2_CT_*x*_, Mo_2_CT_*x*_, V_2_CT_*x*_, Ti_3_C_2_T_*x*_, Ti_3_CNT_*x*_, Mo_2_TiC_2_T_*x*_, Mo_2_Ti_2_C_3_T_*x*_, Nb_4_C_3_T_*x*_, V_4_C_3_T_*x*_, Ta_4_C_3_T_*x*_, and Mo_4_VC_4_T_*x*_ were analyzed with FTIR spectroscopy. Multiple methodologies were evaluated. The metallic nature and greatly varying IR absorptivity of MXenes give them low IR transmittance.^10^ Therefore, the KBr (potassium bromide) method with applied and concave rubberband correction was selected as the best-suited technique (SI: Sections #1 and #2). The advantages of KBr with concave rubberband correction over attenuated total reflectance (ATR) are highlighted in Figure S1.

MXenes belong to the D_3d_ point group^13^ and their vibrations are expressed in Mulliken symbols (eq 1), where *N* represents the total number of atoms in the structure. E_u_ and A_2u_ are IR-active modes, as shown with irreducible representations theory (eq 2), while Γ_optical_ represents the symmetry species of optical excitation in a D_3d_ point group. To the best of our knowledge, no first-principles study dedicated to FTIR spectroscopy of MXenes has been published to date. Available data are limited and highlight IR-active modes alongside Raman-active modes.^39−44^
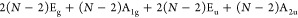
1
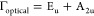
2

E_g_ Raman-active and E_u_ IR-active modes are doubly degenerate. E_u_ mode stands for in-plane movements and is consistent with stretching, while A_2u_ stands for out-of-plane movements, consistent with bending.^45^ In-plane vibrations can be divided into either symmetric when all atoms stretch in the same direction, or antisymmetric,^27^ with atoms stretching in opposite directions.^46,47^ Out-of-plane modes represent wagging when atoms with the same surface charge sign are involved or twisting when atoms’ surface charge signs are opposite.^46,47^

In Ti_3_C_2_T_*x*_ (M_3_X_2_T_*x*_ structure type), the middle-layer region (the inner Ti layer) does not always remain inert, yielding multiple vibration modes (Figure 3a), as reported elsewhere.^13,45−47^ It is in agreement with computational data provided in Table 1, such as E_u_ (Ti–C) symmetric stretching, E_u_ (C–T_*x*_) symmetric stretching, E_u_ (C–T_*x*_) antisymmetric stretching, A_2u_ (Ti–T_*x*_) wagging (bending), A_2u_ (T_*x*_) twisting (bending), and A_2u_ (C–C) twisting (bending). Theoretical analysis (eqs 1 and 2) suggests more vibrational modes than the experimentally observed data. Here, we focus on the most prominent modes corresponding to the experimentally observed bond vibrations.^17,18^ It is important to note that additional modes predicted in Table 1 could potentially exhibit degeneracy (i.e., have the same cutoff frequency (Figure S2) but different field configurations), resulting in overlapping wavenumber ranges (Tables 1 and S1).

**Table 1:**
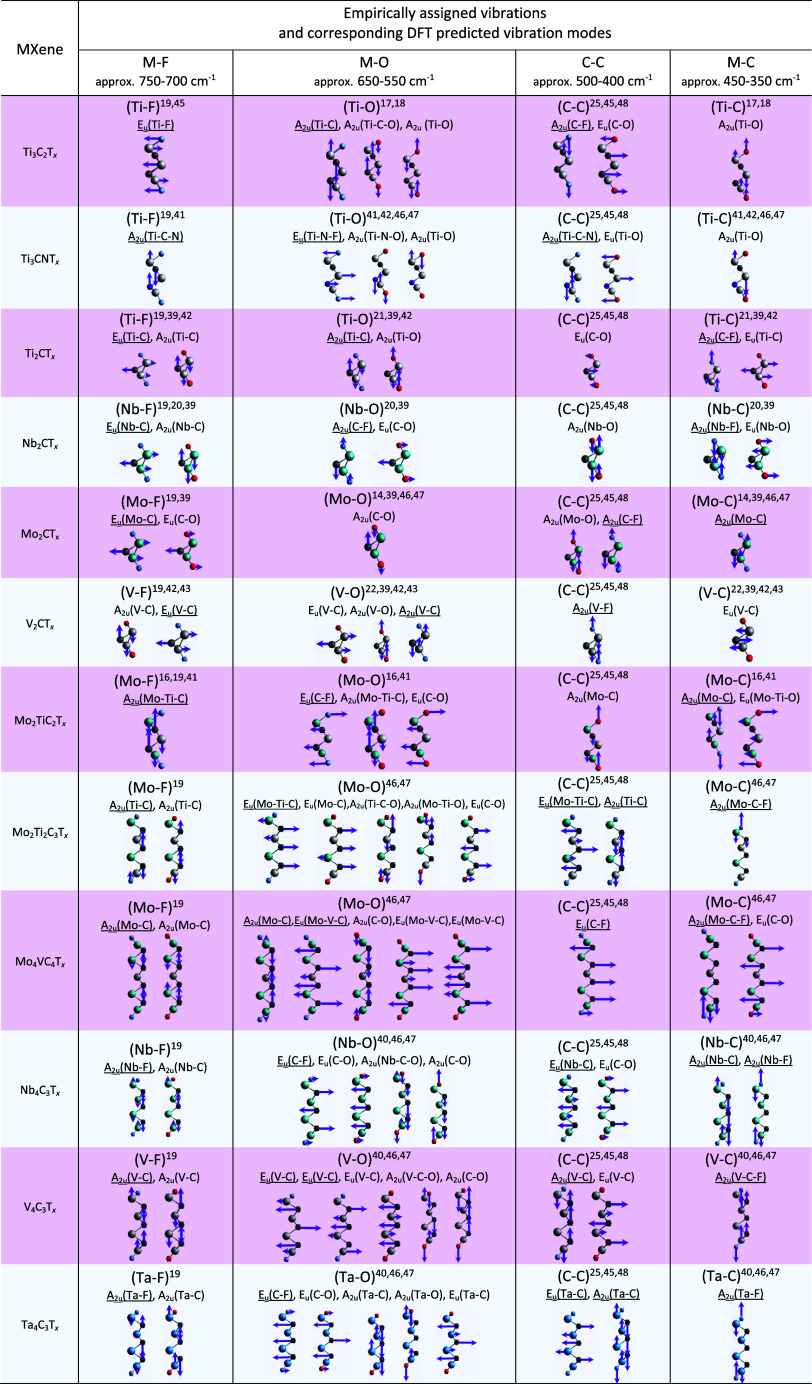
Empirically Assigned^16−18,20−22^ Vibrations vs. DFT-Predicted Vibration Modes^a^

a Empirical T_*x*_ is provided in brackets, DFT-predicted T_*x*_ = F_2_ is underlined, and T_*x*_ = O_2_ is not underlined. DFT positions are selected as the closest match to the empirical range since DFT positions do not always match the empirical range precisely. Discrepancy is expected as we use a single terminations model; moreover, FTIR is always provided in a broad range empirically. All images were generated in VESTA.

FTIR peak positions are provided in Figure 3b, and FTIR peak assignments are shown in Figure 3c. Ti_3_C_2_T_*x*_ spectrum has two major regions: the 4000 to 1400 cm^–1^ region has FTIR peaks corresponding to confined water,^23^ including O–H stretching at 3600–3200 cm^–1^ and −OH surface terminations resulting in O–H bending^45^ at 1500–1300 cm^–1^, as well as carbon bond vibrations,^17,18^ including C–H stretching at 3000–2800 cm^–1^, C=O stretching at 1750–1700 cm^–1^, C–O stretching at 1700–1550 cm^–1^, and C–H bending at 1500–1400 cm^–1^. When the water content in Ti_3_C_2_T_*x*_ decreased (Figure S3), the O–H stretching vibration at 3600–3200 cm^–1^, unlike carbon vibrations, decreased in intensity.^23^ This was achieved via annealing (e.g., heating in a vacuum oven) or specific synthesis routes, like using molten salts.^37^ The vibration peaks in the 1700–1550 cm^1^ range arise from overlapping contributions of C–O stretching and O–H bending vibrations^24^ (as shown in Figures S2, S3, and Table 1). However, in MXenes, the C–O stretching vibration corresponding to the E_u_ (C–T_*x*_) vibrational mode dominates^17,18,41,45^ the 1700–1550 cm^1^ spectral range. Therefore, a 1700–1550 cm^1^ peak can be primarily assigned to C–O stretching.

The second part of the spectrum is the fingerprint region from 1400 to 400 cm^–1^, with the following bond vibrations observed: carbon–fluorine C–F^17,18^ stretching at 1400–1000 cm^–1^, titanium–fluorine Ti–F^19,45^ bending at 750–700 cm^–1^, titanium-oxide Ti–O^17,18^ bending at 650–550 cm^–1^, and titanium carbide Ti–C^17,18^ stretching at 450–350 cm^–1^. A C–C^45,48^ bending vibration at 500–400 cm^–1^ is observed within the fingerprint region. However, the C–C vibration is a weak A_2u_ (C–C) IR mode and, therefore, is not helpful for FTIR identification of Ti_3_C_2_T_*x*_. The C–F bond vibration in Ti_3_C_2_T_*x*_ originates from fluorine surface terminations at the defect sites (Ti vacancies) and flake edges. FTIR spectroscopy can easily detect it due to this technique’s sensitivity to the C–F vibrations.

In complex systems like Ti_3_C_2_T_*x*_,^17−19,23^ the coordination environment (the interaction between neighboring bonds) affects the vibration fingerprint, causing shifts in FTIR peak positions. Ti_3_C_2_T_*x*_ shows the blue shift of C–O stretching and the red shift of Ti–F bending vibrations (Figure 3b,c). C–O stretching vibration blueshifts from its regular position^25,26^ at 1300–1100 cm^–1^ to the 1700–1550 cm^–1^ range.^17,18^ The blue shift is present due to the confined water contained within TI_3_C_2_T_*x*_.^23^ A metal–fluorine bending vibration Ti–F redshifts from its regular position in the 1000–850 cm^–1^ range^25,26,46,47^ to 750–700 cm^–1^.^19,45^ This is due to the simultaneous presence of titanium, oxygen, and fluorine in the surface layer of Ti_3_C_2_T_*x*_.^19^

The experimentally recorded FTIR spectra for 12 selected MXenes are provided in Figure 4. The experimental data correlate well with DFT-predicted values, which will be discussed later. Figure 4 shows the fingerprint region with clear differences among various MXenes. The additional 4000–1400 cm^–1^ range data are provided in Figure S4. Each MXene in this range contains the following vibrations: O–H^16,17,20−23^ stretching at 3600–3200 cm^–1^, C–H^16,17,20−22^ stretching at 3000–2800 cm^–1^, C=O^16,17,20−22^ stretching at 1750–1700 cm^–1^, C–O^16,17,20−22^ stretching at 1700–1550 cm^–1^, C–H^16,17,20−22^ bending at 1500–1400 cm^–1^, and O–H^16,17,20−23^ bending at 1500–1300 cm^–1^. In Figure 4, M is Ti, Nb, V, Mo, or Ta. For all 12 selected MXenes, the following vibrations are observed within the fingerprint region: carbon–fluorine C–F^16,17,20−22^ stretching at 1400–1000 cm^–1^, metal–fluorine M–F^19,39,41−43,45^ bending at 750–700 cm^–1^, metal oxide M–O^16,17,20−22,39,45^ bending at 650–550 cm^–1^, weak carbon–carbon C–C^39,45,48^ bending at 500–400 cm^–1^, and metal carbide M–C^17,18,20,39,45^ stretching at 450–350 cm^–1^.

Figure 4 shows the correlation between the FTIR peak positions and the chemical composition of MXene. For example, additional vibrations appear in carbonitrides, such as Ti_3_CNT_*x,*_ and mixed metal MXenes, such as Mo_2_TiC_2_T_*x*_, Mo_2_Ti_2_C_3_T_*x*_, and Mo_4_VC_4_T_*x*_. In Ti_3_CNT_*x*_, the C–N^26^ stretching is at 1342–1266 cm^–1^ range. In mixed metal MXenes, the higher electronegativity metals M_1_ with a higher affinity to attract shared electrons form the following bond vibrations: M_1_–F bending at 750–700 cm^–1^, M_1_–O bending at 650–550 cm^–1^, and M_1_–C stretching at 450–350 cm^–1^. At the same time, lower electronegativity metals M_2_ form additional M_2_–O^16,22,39^ stretching bond vibrations at 900–750 cm^–1^ range. Metal oxide stretching modes are expected to vibrate in the 400–200 cm^–1^ range,^25,26,46,47^ as stretching of ionic M–O bonds requires more energy than bending due to disruption of its electrostatic attraction. However, the coordination environment of mixed metal MXenes causes a blue shift of M_2_–O stretching from its regular position at 400–200 to 1000–750 cm^–1^.

**Figure 5:**
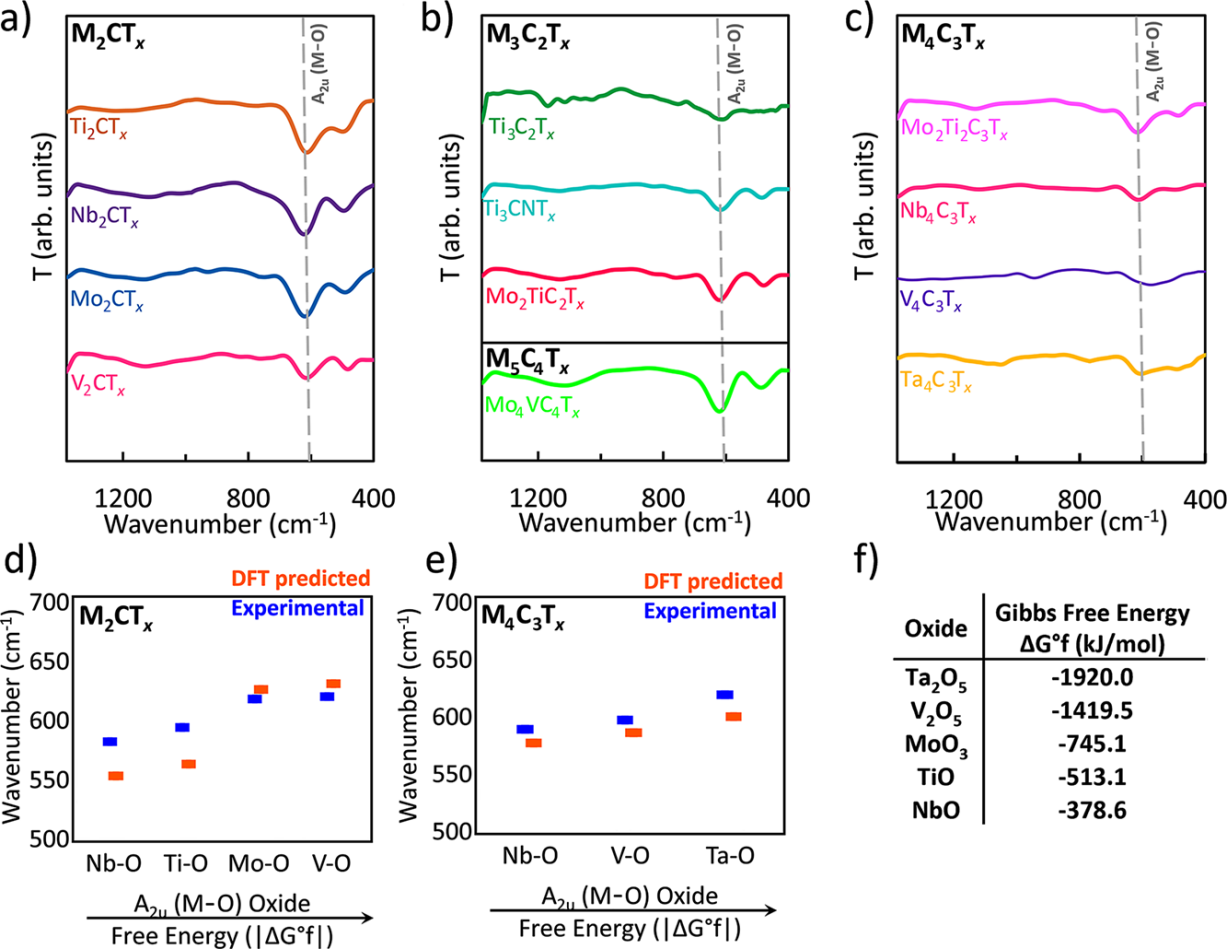
Trends in spectral behavior depending on MXene structure type for (a) M_2_CT_*x*_, (b) M_3_C_2_T_*x*_ and M_5_C_4_T_*x*_, (c) M_4_C_3_T_*x*_, where the dependence of experimental and DFT-predicted A_2u_ (M–O) vibration mode vs its corresponding oxide absolute value of free energy formation (|Δ*G*°_f_|) is highlighted for (d) M_2_CT_*x*_, and (e) M_4_C_3_T_*x*_. The comparison of free energy values for each corresponding oxide is provided in (f). T is transmittance.

In Figure 5, fingerprint region profiles are provided for various MXene structures. Figure 5a shows the M_2_CT_*x*_ structure type, Figure 5b shows the M_3_C_2_T_*x*_ and M_5_C_4_T_*x*_ structures, and Figure 5c shows the M_4_C_3_T_*x*_ structures. The A_2u_ (M–O) is the most intense IR mode of the fingerprint region as M–O bending vibrations at 650–550 cm^–1^ show the highest intensity for all selected MXenes, leading to an ease of detection. The high polarity of M–O bonds and their relatively high content^49^ can explain this.^46,47^ With an increase in the number of layers, the A_2u_ (M–O) bending vibration becomes less defined. For example, M–O peaks are much broader for M_4_C_3_T_*x*_ (Figure 5c) than for M_2_CT_*x*_ (Figure 5a). It may be caused by stacking interactions, where with the increase in the number of layers/atoms in the structure, M–O bonds start to interact with one another (van der Waals forces), leading to splitting^50^ of FTIR peak vibrations, which causes broadening, which also correlates greatly with DFT-predicted values in Figure 4. The position of the A_2u_ (M–O) vibration mode in MXenes is influenced by the thermodynamic stability of the corresponding metal oxide. Figure 5d,e compares the experimental and DFT-predicted wavenumbers of M–O vibrations for various MXene structures (the entirety of wavenumber positions is provided in Table S1). The position estimates related to the vibration ranges are also provided in Table 1. A trend emerges: MXenes containing transition metals that form oxides with more negative Gibbs free energies of formation (Δ*G*°_*f*_)^51,52^ (Figure 5f) tend to exhibit A_2u_ (M–O) vibrations at higher wavenumbers. Δ*G*°_*f*_ reflects the inherent stability of a compound. A more negative Δ*G*°_*f*_ for a metal oxide indicates a more energetically favorable and spontaneous oxidation process. In simpler terms, metals with a higher propensity to form stable oxides (more negative Δ*G*°_*f*_) tend to have more stable M–O bonds in their corresponding MXenes. This translates to higher energy required to break the oxide bonds, as reflected in the higher wavenumbers (SI: Section #1) observed in the A_2u_ (M–O) mode (Figure 5d,e). This correlation between the | Δ*G*°_*f*_ | and A_2u_ (M–O) vibration positions aligns with the observed oxidation behavior of MXenes. Vanadium (V) and tantalum (Ta)-based MXenes, for example, are known to be more susceptible to oxidation,^53,54^ which is beneficial for electrochemical redox reactions. This can be explained by their corresponding oxides having significantly more negative Δ*G*°_*f*_ values, indicating a more potent thermodynamic driving force for oxidation. Consequently, the M–O bonds in these MXenes exhibit higher wavenumbers in the A_2u_ (M–O) mode, reflecting their more favorable bond character. Though FTIR struggles with directly probing metal–ligand vibrations, we employed literature analysis and DFT calculations to assign M–O and M–C modes. Notably, for MXene characterization, FTIR excels in analyzing crucial O–H vibrations and offers a highly sensitive surface functionalization technique for fingerprint region analysis.

Experimental observations often reveal variation and overlap in wavenumber positions of the FTIR peaks. Without means to calculate it, such as DFT, the overall shape of the spectra (e.g., FTIR peak width and relative intensity, as discussed above) becomes the only leverage for analyzing the results. On the other hand, DFT-based calculations can theoretically predict phonon dispersions, based on which the wavenumber of each IR-active vibration mode can be explicitly derived, thus providing a theoretical basis for a better interpretation of experimental data. Both −F and =O terminations in the current work were selected to occupy a top site (above the lower layer metal, Figure 2b) in MXene unit cells (space group *P*6_3_/*mmc*, and herringbone-type^201^ structure for Mo_4_VC_4_T_*x*_). Corrected phonon band dispersions are provided in Figure S2. Generally, DFT predicts IR vibrations for ideal MXene crystal structures, where lattice defects and disorder are not considered. Despite those limitations, comparing DFT predictions to experimental FTIR spectra helps to explain the MXene profiles. As seen in Figure 4, the increased number of atoms in the primitive unit cell of the DFT model raises the number of predicted modes due to vibrational degrees of freedom increase, correlating with broader peaks observed for MXene structures (Figure 5a–c) of higher atomic layer count (M_2_CT_*x*_ < M_3_C_2_T_*x*_ < M_4_C_3_T_*x*_ < M_5_C_4_T_*x*_). MXene samples often possess a mix of different terminations (denoted as T_*x*_). This contrasts with the DFT calculations, which use idealized crystal structure models with pure −F or =O terminations. As a result, the predicted IR peak positions from DFT might not perfectly match the experimental data. While the discrepancy exists, it can be used to our advantage for interpreting the experimental data with the help of DFT predictions. In Figure 4 and Table 1, the experimental vibrational peaks often fall somewhere between the DFT-predicted peaks’ overlapping regions for −F and =O terminations. This suggests that the experimental vibrations represent the average of these two idealized scenarios. This discrepancy is typically within 100 cm^–1^ (Table S1), which is an excellent agreement considering the model’s limitations. Additionally, FTIR peak positions are inherently reported within a range of a few hundred cm^–1^ (SI: Section #1). The observed discrepancy highlights the importance of mixed terminations in real MXenes.

Table 1 compares the empirically assigned FTIR peak vibrations of Ti_2_CT_*x*_, Nb_2_CT_*x*_, Mo_2_CT_*x*_, V_2_CT_*x*_, Ti_3_C_2_T_*x*_, Ti_3_CNT_*x*_, Mo_2_TiC_2_T_*x*_, Mo_2_Ti_2_C_3_T_*x*_, Nb_4_C_3_T_*x*_, V_4_C_3_T_*x*_, Ta_4_C_3_T_*x*_, and Mo_4_VC_4_T_*x*_ with the vibration modes predicted by DFT calculations (the positions are provided as estimates to the four selected vibration ranges, namely, M–F, M–O, C–C, and M–C modes), where the entire range of vibrations (experimental vs DFT) is provided in Table S1. It reveals the dominance of M–T_*x*_, C–T_*x*_, and M–C vibration modes. This correlates well with the empirical vibrations’ assignment. The significance of the predicted IR modes lies in their ability to refine the assignment of empirical vibration peaks. Traditionally, such assignments heavily rely on literature reviews of similar materials. However, with extremely limited information about the IR spectra of MXenes in the literature, our work emphasizes the importance of theoretical predictions for more reliable assignments. While empirically assigned as the most intense (M–O) vibration (around 650–550 cm^–1^), DFT calculations reveal a more complex picture. The wavenumber range corresponding to the empirically assigned M–O vibration exhibits the highest density of DFT-predicted vibrational modes compared to those of other spectral regions. This overlap originates from the molecular perspective, where multiple vibrational modes can contribute to a single observed peak position. Empirically, this translates to a broad and intense peak, reflecting the combined contributions of these overlapping modes as opposed to single, isolated vibrations.

DFT calculations of the selected surface terminations (−F and =O) provide vibrational mode predictions for the specific MXenes under study. The high density of predicted modes in the M–O peak region strengthens the assignment by suggesting that multiple vibrational motions involving the metal and oxygen atoms contribute to the observed peak. This insight would not be readily apparent from just literature comparisons, highlighting the power of DFT calculations in unraveling the complexities of FTIR spectra for novel materials like MXenes. Including mixed terminations in future DFT models could improve the accuracy of the predictions. Additionally, structure disorder possesses certain challenges. The effectiveness of FTIR for double-M in-plane and out-of-plane ordered and disordered MXenes varies. Ordered structures with long-range interactions show sharp FTIR peaks, while disordered structures exhibit broader peaks due to a lack of long-range interactions. Complementary techniques such as XRD, XPS, or Raman spectroscopy may be needed for the analysis of disordered double-M MXenes. The ability of FTIR spectroscopy to directly quantify the ordering in double-M MXenes is limited. However, it can provide indirect insights into the ordering based on the characteristics (full width at half-maximum) of the observed peaks. For this study, which focused on establishing general trends in FTIR spectra of MXenes, we limit empirical analysis and DFT to −F and =O terminations.

## Conclusions

This work provides a unified approach to the FTIR spectroscopy analysis of MXenes. We have provided recommendations for recording and interpreting the IR spectra of MXenes. FTIR spectroscopy was used to analyze 12 MXenes – Ti_2_CT_*x*_, Nb_2_CT_*x*_, Mo_2_CT_*x*_, V_2_CT_*x*_, Ti_3_C_2_T_*x*_, Ti_3_CNT_*x*_, Mo_2_TiC_2_T_*x*_, Mo_2_Ti_2_C_3_T_*x*_, Nb_4_C_3_T_*x*_, V_4_C_3_T_*x*_, Ta_4_C_3_T_*x*_, and Mo_4_VC_4_T_*x*_ – demonstrating the influence of MXene composition, structure, and surface terminations on their characteristic fingerprints. The empirical FTIR peak assignment was supported with DFT predictions using VASP and PBE functional approximations for −F and =O surface-terminated MXenes. The following vibration modes were assigned to the most widely used Ti_3_C_2_T_*x*_ MXene: E_u_ (Ti–C) symmetric stretching, E_u_ (C–T_*x*_) symmetric 400, E_u_ (C–T_*x*_) antisymmetric stretching, A_2u_ (Ti–T_*x*_) wagging (bending), A_2u_ (T_*x*_) twisting (bending), and A_2u_ (C–C) twisting (bending). The correlation between experimental and theoretical data allowed for the assignment of FTIR peak vibrations for the possible bonds in the MXene spectra. DFT calculations predict a significantly higher density of vibrational modes within the wavenumber range corresponding to the observed M–O vibration peak. Peak assignment for all selected MXenes was completed in the fingerprint region. The changes in the FTIR spectra depending on the number of layers in the MXene structure were investigated. With the increase of the number of layers, where M_2_CT_*x*_ < M_3_C_2_T_*x*_ < M_4_C_3_T_*x*_ < M_5_C_4_T_*x*_, the A_2u_ (M–O) bending vibration becomes less defined, with M–O peaks being much broader for M_4_C_3_T_*x*_ than for M_2_CT_*x*_. The A_2u_ (M–O) mode position also depends on its corresponding oxide Gibbs free energy of formation (Δ*G*°_f_). The wavenumber position is directly proportional to the absolute value |Δ*G*°_*f*_|. This work establishes the foundation for the FTIR analysis of MXenes, provides reliable recommendations, and demonstrates the use of an easy and dependable FTIR technique for the analysis of MXenes.

## Materials and Methods

### Materials

MXenes were synthesized^28,29^ from corresponding MAX phase precursors via the wet-chemical top-down route (Table 2). MAX phases^5,10,30−34^ were sintered from commercial powders.

**Table 2:** Synthesis Conditions of MAX Phases & MXenes^a^

MAX	molar ratio	sintering	etchant mL/1g MAX	etching	delamination per 1g MAX
Ti_3_AlC_2_	TiC:Al:Ti=2:2.2:1.25	1400°C,2h	HCl:H_2_O:HF=12:6:2	24h,35°C	1g LiCl,20mL H_2_O,12h,35°C
Ti_3_AlCN	Ti:AlN:C=3:1:1	1500°C,2h		24h,35°C	
Ti_2_AlC	Ti:Al:C=2:1.1:0.9	1550°C,2h		12h,25°C	
Nb_2_AlC	Nb:Al:C=2:1.1:0.9	1550°C,2h	HCl:HF=12:8	48h,50°C	5mL TMAOH,15mL H_2_O,12h,35°C
Mo_2_Ga_2_C	Mo_2_C:Ga=1:8	850°C,15d	20mL HF	48h,50°C	
V_2_AlC	V:Al:C=2:1.1:0.9	1550°C,2h	HCl:HF=12:8	72h,50°C	
Mo_2_TiAlC_2_	Mo:Ti:Al:C=2:1:1.1:2	1600°C,4h	20mL HF	48h,50°C	
Mo_2_Ti_2_AlC_3_	Mo:Ti:Al:C=2:2:1.3:2.7	1600°C,4h	20mL HF	48h,50°C	
Mo_4_VAlC_4_	Mo:V:V_2_O_3_:Al:C=4:0.9:0.05:1.2:3.5	1650°C,4h	20mL HF	192h,50°C	
Nb_4_AlC_3_	Nb:Al:C=4:1.1:2.7	1650°C,4h	HCl:HF=12:8	168h,50°C	
V_4_AlC_3_	V:V_2_O_3_:Al:C=4:0.05:1.5:3	1500°C,2h	HCl:HF=12:8	192h,50°C	
Ta_4_AlC_3_	Ta:Al:C=4:1.5:3	1500°C,6h	10mL HF	24h,35°C	

a For all MAX phases, the sintering ramp rate was 3 °C/min.

The materials were purchased as follows: Titanium (99.5% Thermo Fisher Scientific, −325 mesh), Titanium Carbide (99.7% Sigma-Aldrich), Aluminum Nitride (98% Sigma-Aldrich), Niobium (99.8% Thermo Fisher Scientific, −325 mesh), Tantalum (99.9% Thermo Fisher Scientific, −325 mesh), Gallium (99.9% Sigma-Aldrich), Molybdenum (99.9% Thermo Fisher Scientific, −250 mesh), Vanadium (99.5% Thermo Fisher Scientific, −325 mesh), Vanadium-III Oxide (98% Sigma-Aldrich), Aluminum (99.5% Thermo Fisher Scientific, −325 mesh), Graphite (99% Thermo Fisher Scientific, −325 mesh), Hydrochloric Acid (38 wt.% aq. Thermo Fisher Scientific), Hydrofluoric Acid (51 wt.% aq. Thermo Fisher Scientific), Lithium Chloride (LiCl, 99% anhydrous, Thermo Fisher Scientific, −20 mesh), and Tetramethylammonium Hydroxide (TMAOH, 25 wt % aq. Sigma-Aldrich). Sintering of MAX phases was performed in a tube furnace (Carbolite Gero). Etching was performed by using a hot plate (Corning, Sigma-Aldrich). After etching, MXenes were washed with DI (deionized) water via centrifugation (Thomas Scientific Sorvall ST 40) at 3500 rpm until achieving a neutral pH. Delamination was performed using either LiCl or TMAOH. After delamination, the LiCl/TMAOH was discarded via centrifugation, followed by subsequent collection cycles. Delaminated MXene dispersions were concentrated at 10,000 rpm and processed via vacuum-assisted filtration.

### FTIR

Bruker Invenio IR Spectrometer was used to record IR spectra in transmittance with additional atmospheric compensation in the range of 4000–400 cm^–1^. The resolution was set to 4 cm^–1^ with a total of 14 scans. Opus software was used to process the data. The smoothing of the spectra was applied with a 25-point average. Baseline correction was performed with the following parameters: concave rubberband correction, 2 iterations, 4 baseline points, exclude CO_2_ peaks (SI: Section #2). Vacuum-filtered MXene was ground manually with KBr (Sigma-Aldrich, Potassium Bromide, BioUltra 99.5%_at_, 119 g/mol) using an agate mortar and pestle (Thomas Scientific, 50 mm diameter). 0.2 g portion of KBr and 0.001 g of MXene were used for each sample. This ratio yielded an approximately 1 mm thick pellet. The sample was ground until all particles were of approximately equal size and had a very fine powder consistency. This prevented IR wave diffraction, which happens when particle sizes vary and can interfere with spectra capture. Following the grinding, a mixture was transferred to a pressing setup (Split Type Dry Pellet Pressing Die Set, MSE Supplies, 12.7 mm diameter). Pellets were pressed at 6 t using a hydraulic press (Carver). The pristine KBr pellet was recorded as a background signal before the MXene/KBr sample pellet recording. The pellets were placed in the sample holder perpendicular to the direction of the IR waves. If multiple samples were prepared, each sample was recorded directly following pressing to minimize interference from moisture absorption. The data were recorded at ambient humidity and temperature.

### DFT

First-principles calculations were performed based on DFT using the Vienna Ab initio Simulation Package (VASP).^55^ The projector-augmented wave (PAW)^38^ method was employed. The generalized gradient approximation (GGA) functional developed by Perdew–Burke–Ernzerhof^55^ was used to describe the exchange-correlation interactions among electrons. To mitigate the potential overestimation of lattice parameters often observed with GGA functionals, we employed a geometry relaxation step prior to further DFT calculations, which improves the accuracy of the selected model. A vacuum layer (∼10 Å) was added along the normal direction of the MXene surface to prevent interactions between the 2D slab and its periodic image. Gaussian smearing was applied for the Brillouin-zone integration. The width of the smearing was 0.05 eV. The energy cutoff of the plane wave basis was 600 eV. A 6 × 6 × 1 Γ-centered grid was used for the k points mesh. The density functional perturbation theory (DFPT) method was used for phonon calculations. 4 × 4 × 1 supercells were selected for phonon calculations. Phonon calculations were processed using Phonopy.^56^ Phonon dispersion bands in Figure S2 are corrected according to the rotational sum rule applying Phyton with the hiPhive package.^38^ Based on the results of phonon calculations, the wave numbers of IR-active vibration modes were analyzed and predicted using the Phonopy Spectroscopy package.^38^

### Optical Imaging

Optical imaging was performed with KEYENCE VK-X1000 using the following setting: 5:1 zoom ratio, laser + LED ring mode, and the width of each image is 22.5 μm.

### XRD

X-ray diffraction was performed with a Rigaku SmartLab (40 kV/30 mA) and MiniFlex (40 kV/15 mA) with Cu K_α_ radiation. The step size of the scan was 0.01° with a step duration of 4 s for MAX phase powders and 0.02° step size with 0.6 s step duration for MXene films.

## Supporting Information Available

FTIR basics; FTIR data processing; FTIR methodologies; spectra of annealed vs vacuum-filtered Ti_3_C_2_T_*x*_ samples; normalized FTIR spectra of MXenes, phonon dispersion bands; and experimental vs DFT-predicted peak positions (PDF)
