# Supplementary material for: Fourier-Transform Infrared Spectral Library of MXenes
Source: Chem Mater. 2024 Aug 21;36(17):8437–46. doi: 10.1021/acs.chemmater.4c01536 (PMC11393797; doi:10.1021/acs.chemmater.4c01536)
Supplement: Supplementary file 1 — cm4c01536_si_001.pdf [file cm4c01536_si_001.pdf]

Fourier-Transform Infrared Spectral Library of MXenes

Tetiana Parker[1,2], Danzhen Zhang[1,2], David Bugallo[2,3], Kateryna Shevchuk[1,2], Marley Downes[1,2], Geetha Valurouthu[1,2], Alex Inman[1,2], Benjamin Chacon[1,2], Teng Zhang[1,2], Christopher E. Shuck[1,4], Yong-Jie Hu[2], Yury Gogotsi[1,2*]

[1]*A.J. Drexel Nanomaterials Institute, Drexel University, 3141 Chestnut St., Philadelphia, PA 19104, US*

[2]*Department of Material Science and Engineering, Drexel University, 3141 Chestnut St., Philadelphia, PA 19104, US*

[3]*Centro de Investigación en Química Biolóxica e Materiais Moleculares (CIQUS), Universidade de Santiago de Compostela, 15782 Santiago, Spain*

[4]*Department of Chemistry and Chemical Biology, Rutgers University, Piscataway, NJ 08854, US*

[*] Corresponding author: Yury Gogotsi gogotsi@drexel.edu

■ SECTION #1 FTIR BASICS

FTIR (Fourier-Transform Infrared) Spectroscopy determines the chemical composition of various materials. The sample is exposed to IR (Infrared) waves for that purpose. The incident waves excite molecules, causing vibration, resulting in IR absorbance/transmittance. One of the applications is identifying unknown substances based on their characteristic absorbance/transmittance spectra. Organic chemists use absorbance vs. wavenumber and inorganic transmittance vs. wavenumber. Meantime, the wavenumber is reversibly proportional to wavelength. Divide 10,000 by wavenumber (cm^{-1}) to obtain wavelength (μm). Further, we will use transmittance to maintain convention, as MXenes are inorganic. Unlike Raman spectroscopy, which uses focused lasers of specific intensities to interact with a sample, Fourier-Transform Infrared (FTIR) spectroscopy relies on a broad infrared (IR) source. A Michelson interferometer (MI) generates this broad IR range in FTIR, with a laser only needed to guide the IR source. The range is near-infrared (12500-4000 cm^{-1}), mid-infrared (4000-400 cm^{-1}), or far-infrared (400-50 cm^{-1}) (NIR, MIR, or FIR respectively). Most spectrometers are suitable to work only in MIR. Upon data processing, the wavenumber (cm^{-1}) must be represented in a reverse unit order, because we start at low and go to high energy (from 4000 down to 400 cm^{-1}). Transmittance is normalized so that the sample's highest transmittance has the same value in each sample, resulting in arbitrary units (a.u.) of measurement. This is because we analyze the wavenumber (cm^{-1}) range at which transmittance occurs and not its value, since the value may vary significantly depending on measurement conditions (e.g., humidity, etc.). The range at which the transmittance spike occurs is specific to each bond vibration and is called the FTIR peak range. The arbitrary transmittance relationship of one peak to another is a unitless value; however, it can be compared as a last resort to identify chemical compounds ratio when otherwise unclear. When analyzing spectra, it is important to remember that transmittance peaks are rarely observed at the same wavenumber (cm^{-1}) because FTIR is extremely sensitive to sample preparation. Therefore, vibrations are always provided in the FTIR as a certain range value, usually a couple of hundred inverse centimeters (cm^{-1}). The vibration range also delivers information on the strength of the corresponding bond. The higher the wavenumber, the lower the wavelength. The lower the wavelength, the higher the vibrational frequency, and so the higher the vibrational energy required to break the bond. MXenes, besides being inorganic, are known IR shielding materials. Therefore, their transmittance is extremely low. Nonetheless, it is still possible to analyze MXenes quite efficiently. To do so, specific sample preparation, an in-depth literature search, and molecular modeling such as DFT (Density Functional Theory) are required.

■ SECTION #2 FTIR DATA COLLECTION AND PROCESSING

FTIR can usually be performed by a few various approaches; one of the ways is using the Attenuated Total Reflectance (ATR) method, where the incident IR (Infrared) wave is reflected through a diamond crystal surface, and the sample is placed directly on contact with the crystal. Among the

advantages of ATR is the possibility of studying liquid solutions alongside solids, and no additional sample preparation is required. However, this technique is not sensitive enough for MXene samples, as they are inorganic and exhibit extremely low transmittance, which is insufficient to be properly detected *via* ATR. Therefore, in this work, the KBr method is used instead. This method requires pellet pressing using a manual ground mix of sample and IR transparent matrix holder, potassium bromide (KBr), which gives the method its name. This method is beneficial during transmittance measurements as it allows IR to pass directly through the sample, and it is very suitable for MXenes, as it increases the sensitivity of recorded data. Besides specific sample preparation, MXene spectra require baseline correction, which will allow for the peaks to become prominent. Concave rubberband correction is a baseline correction method that adjusts data to remove unwanted baseline offsets from the spectral signal. The approach to performing the correction is to determine the minimum convex hull in the data (the minimum area where the majority of a given set of points is accumulated) and subtract it from the spectral range. The correction is usually performed with spectroscopic software and not manually. The actual mechanism is more complicated and can involve multiple algorithms. The general equation is provided below:

$$y_{corrected} = y - y_{min.\ convex\ hull}$$

where y is the original spectral data, $y_{\text{corrected}}$ is the corrected spectral data, and $y_{\text{min. convex hull}}$ is the minimum convex hull of the spectral data. After correction, the data can be saved in both data format (.dat) and Opus format (.0). Data format can later be used to plot spectra in various software, such as Origin, Excel, etc. Exported data undergo normalization and manual peak assignment using literature sources and DFT.

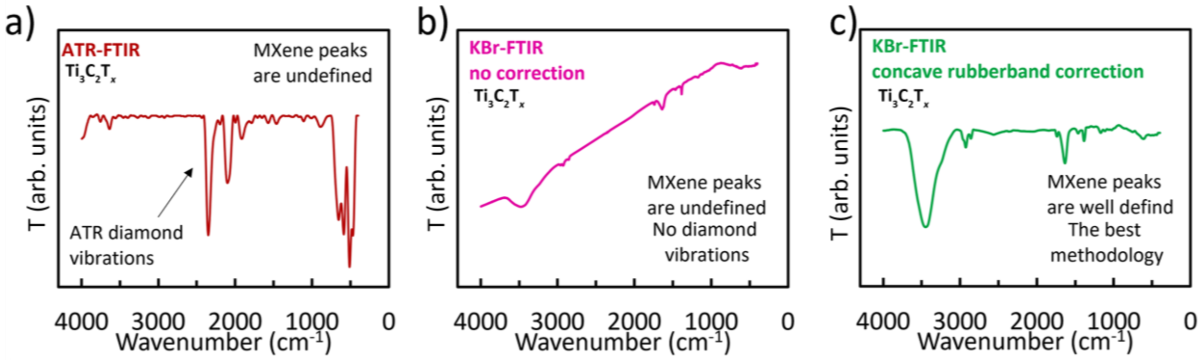

Figure S1. Comparison of various FTIR methodologies. (a) ATR-FTIR, (b) KBr-FTIR with no correction, (c) KBr-FTIR with concave rubberband correction applied. T is transmittance.

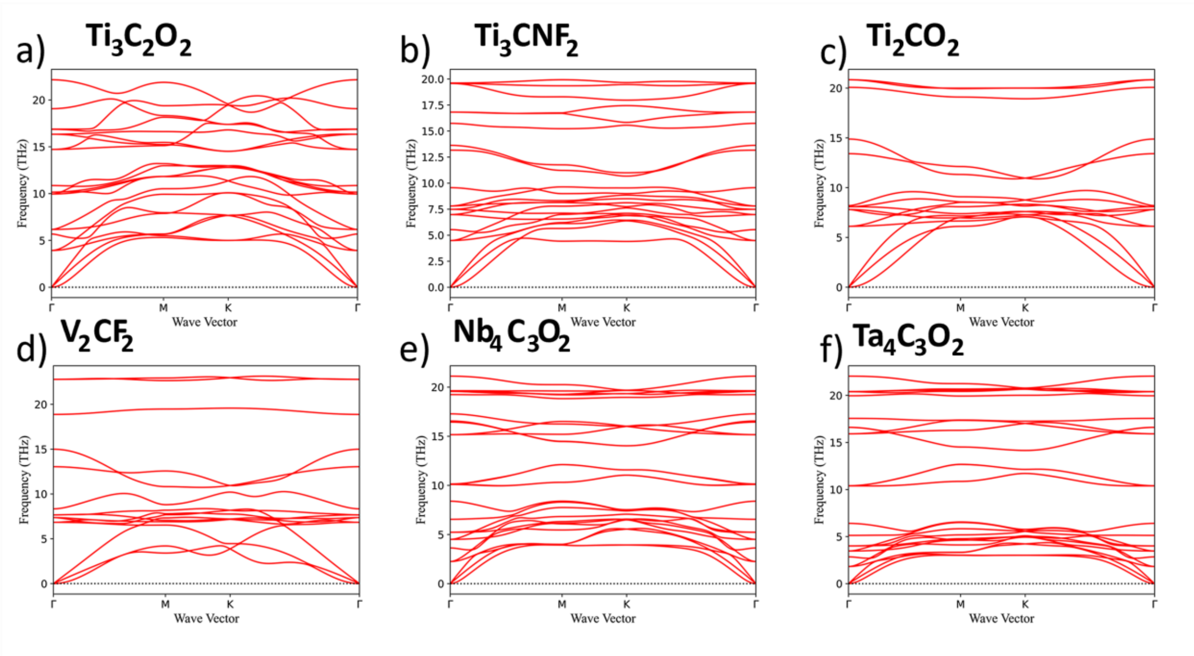

Figure S2. Selected phonon dispersion bands. (a) $Ti_3C_2O_2$, (b) Ti_3CNF_2, (c) Ti_2CO_2, (d) V_2CF_2, (e) $Nb_4C_3O_2$. The DFT-predicted bands are selected to represent the main MXene structure types with various terminations T_x (-F, =O).

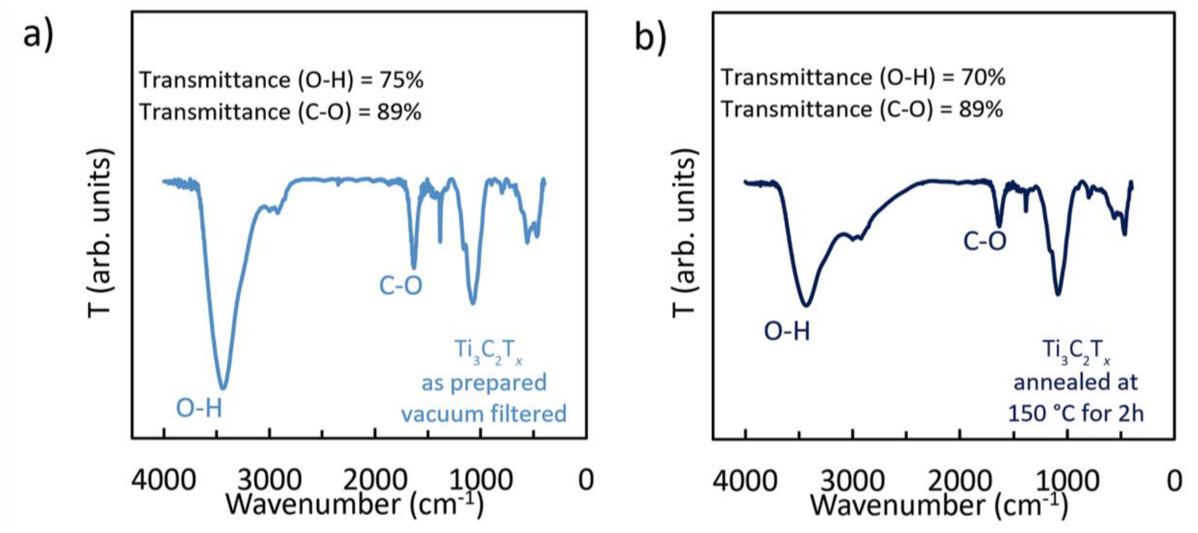

Figure S3. Comparison of $Ti_3C_2T_x$ spectra depending on confined water content. (a) spectrum before annealing and (b) after. T is transmittance.

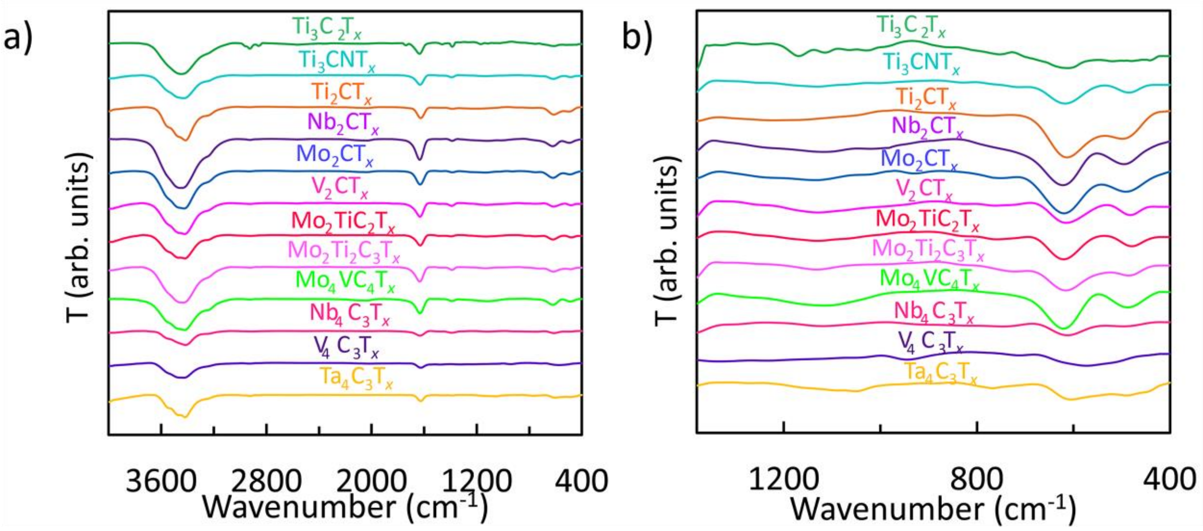

Figure S4. Normalized MXenes FTIR in (a) 4000-400 cm^{-1} range and (b) 1400-400 cm^{-1} range. T is transmittance.

Table S1. The experimental (T_x) vs. DFT-predicted (-F, =O) peak positions in the fingerprint region for Ti_2CT_x, Nb_2CT_x, Mo_2CT_x, V_2CT_x, $Ti_3C_2T_x$, Ti_3CNT_x, $Mo_2TiC_2T_x$, $Mo_2Ti_2C_3T_x$, $Nb_4C_3T_x$, $V_4C_3T_x$, $Ta_4C_3T_x$, and $Mo_4VC_4T_x$.

Structure	Peak positions (cm^{-1}) (**E_u is bold** and *A_{2u} is italic*, T_x is underlined)
$Ti_3C_2T_x$ experimental	1169, 859, 746, 625, 476, 403
$Ti_3C_2F_2$ calculated	**644**, *591*, *464*, *340*, **278**, **227**
$Ti_3C_2O_2$ calculated	*636*, *563*, **491**, *362*, **332**, **206**
Ti_3CNT_x experimental	1250, 1126, 713, 613, 484, 412
Ti_3CNF_2 calculated	*654*, **561**, *525*, *319*, **260**, **233**
Ti_3CNO_2 calculated	*594*, *560*, **456**, *348*, **227**, *196*
Ti_2CT_x experimental	1223, 773, 593, 491, 405
Ti_2CF_2 calculated	**695**, *670*, *447*, **260**
Ti_2CO_2 calculated	*727*, *562*, **527**, **366**
Nb_2CT_x experimental	1124, 770, 581, 496, 417
Nb_2CF_2 calculated	**736**, *552*, *386*, **222**
Nb_2CO_2 calculated	*691*, **632**, *543*, **365**
Mo_2CT_x experimental	1128, 756, 617, 487, 405
Mo_2CF_2 calculated	**716**, *426*, *341*, **203**
Mo_2CO_2 calculated	**732**, *625*, *508*, **306**
V_2CT_x experimental	1124, 754, 619, 480, 405
V_2CF_2 calculated	**760**, *630*, *435*, **246**
V_2CO_2 calculated	*731*, **632**, *586*, **377**
$Mo_2TiC_2T_x$ experimental	1137, 804, 713, 615, 480, 401
$Mo_2TiC_2F_2$ calculated	*641*, **633**, *446*, *264*, **226**, **195**
$Mo_2TiC_2O_2$ calculated	*639*, **589**, *555*, **371**, *260*, **154**
$Mo_2Ti_2C_3T_x$ experimental	1136, 825, 717, 619, 476, 406
$Mo_2Ti_2C_3F_2$ calculated	*676*, **662**, **537**, *496*, *429*, **224**, **208**, **174**
$Mo_2Ti_2C_3O_2$ calculated	*713*, **664**, *563*, *548*, **526**, **347**, *222*, **133**
$Mo_4VC_4T_x$ experimental	1099, 779, 761, 639, 482, 400
$Mo_4VC_4F_2$ calculated	*732*, *652*, **543**, **474**, *429*, *246*, **199**, **144**, *97*
$Mo_4VC_4O_2$ calculated	*696*, *612*, **542**, **520**, **351**, *306*, *188*, **131**, *93*
$Nb_4C_3T_x$ experimental	1120, 723, 588, 482, 408
$Nb_4C_3F_2$ calculated	*648*, **604**, **477**, *425*, *399*, *209*, **161**, **128**
$Nb_4C_3O_2$ calculated	*704*, **654**, *576*, *548*, **506**, **337**, *218*, **150**
$V_4C_3T_x$ experimental	1000, 703, 596, 507, 401
$V_4C_3F_2$ calculated	*656*, **627**, **574**, *481*, *443*, *258*, **196**, **187**
$V_4C_3O_2$ calculated	*670*, **643**, *585*, *564*, **528**, **327**, **219**, **72**
$Ta_4C_3T_x$ experimental	1047, 703, 618, 507, 401
$Ta_4C_3F_2$ calculated	*680*, **662**, **505**, *449*, *373*, *166*, **133**, **91**
$Ta_4C_3O_2$ calculated	*735*, **681**, *586*, *554*, **531**, **347**, *171*, **116**
